# Biomechanical evaluation of biodegradable zinc and polylactide pins for radial head fracture fixation: a human cadaver study

**DOI:** 10.1186/s13018-026-06964-4

**Published:** 2026-05-20

**Authors:** Julian P. Maier, Leon Pohl, Jonas Eck, Benjamin Erdle, Nils Mühlenfeld, Kilian Reising, Michael Seidenstuecker, Hagen Schmal, Ferdinand C. Wagner

**Affiliations:** 1 Department of Orthopedics and Trauma Surgery, University Medical Center Freiburg, Hugstetter Str. 55, 79106 Freiburg, Germany; 2 G.E.R.N Tissue Replacement, Regeneration & Neogenesis, Department of Orthopedics and Trauma Surgery, University Medical Center Freiburg, Freiburg, Germany; 3 Faculty of Medicine, University of Freiburg, Freiburg, Germany; 4 Department of Orthopedic Surgery, University Hospital Odense, Odense, Denmark

**Keywords:** Radial head fracture, Biodegradable implants, Zinc pins, Polylactide pins, Human cadaver, Biomechanics

## Abstract

**Background:**

Displaced or unstable radial head fractures are commonly treated with fragment-preserving fixation to restore joint congruity, maintain stability, and allow early mobilization. Biodegradable implants have become attractive alternatives to metallic implants. However, polymer-based pins may be limited by inferior mechanical strength and a higher risk of secondary loss of reduction. Recently, zinc pins have emerged as a potential alternative, but clinical data and biomechanical testing in human bone models are scarce. This study aimed to compare the biomechanical performance of zinc pins (ZP) and polylactide pins (PP) for the fixation of Mason type II radial head fractures using a human cadaver model.

**Methods:**

Sixteen human cadaver radii from eight donors were used to create standardized Mason type II radial head fractures. Paired left and right radii from each donor were randomly assigned to fixation with either two 2.0 mm ZP or PP. After fracture fixation, specimens underwent cyclic transverse loading (10 cycles), followed by cyclic axial loading (1,000 cycles) between 15 and 50 N at 0.1 Hz, and final load-to-failure testing with continuously increasing axial load (2 N/s). Construct stiffness, micromotion at the fracture site, axial migration (implant loosening), as well as failure loads at 2 mm displacement and construct failure were recorded. A paired statistical analysis was performed to account for donor-specific variability.

**Results:**

Fixation with ZP demonstrated higher construct stiffness (0.77 ± 0.21 vs. 0.33 ± 0.05 kN/mm; p-adj. = 0.028) compared to PP constructs under axial load. Further, lower micromotion at the fracture site (0.05 ± 0.01 vs. 0.10 ± 0.02 mm; p-adj. = 0.042) and higher resistance to progressive loading with higher loads at construct failure were measured in ZP compared to PP (190 ± 44 vs. 116 ± 62 N; p-adj. = 0.042). However, no differences were observed between groups in axial migration (0.36 ± 0.08 mm vs. 0.36 ± 0.08 mm) during cyclic testing, indicating comparable resistance to implant loosening. Notably, construct stiffness under transverse loading and the loads required to reach a clinically relevant fracture displacement of 2 mm (161 ± 37 vs. 112 ± 60 N; p-adj. = 0.116) did not differ between ZP and PP constructs, suggesting adequate stability of both constructs within functional loading ranges.

**Conclusion:**

In this cadaveric biomechanical study, zinc pins provided superior overall mechanical stability compared with polylactide pins for fixation of Mason type II radial head fractures, suggesting they are a promising alternative to the widely used polylactide pins. Importantly, polylactide pins also demonstrated sufficient primary stability without increased implant loosening or reduced resistance to clinically relevant fracture displacement. However, based on our data, zinc pins demonstrated favorable biomechanical performance in terms of construct stiffness and load to failure, offering a greater mechanical safety margin. Further clinical studies are required to evaluate in vivo performance and long-term outcomes of zinc-based biodegradable implants.

## Introduction

Radial head fractures are among the most common injuries of the elbow, accounting for approximately one-third of all elbow fractures [1]. These fractures play a key role in maintaining elbow stability, forearm rotation, and load transmission across the radiocapitellar joint, making accurate classification and appropriate treatment essential to prevent long-term functional impairment [2–4]. Typically, nondisplaced and stable or partially displaced fractures can be treated nonsurgically with good to excellent results [5, 6]. However, the treatment of Mason type II fractures, which are characterized by displaced partial articular involvement (> 30%) without comminution, remains a subject of ongoing debate because of the complex balance between restoring joint stability and minimizing long-term complications [7–9]. Significantly displaced, unstable, or even comminuted fractures typically require surgical treatment with open reduction and internal fixation in order to preserve the native radial head and maintain the physiological biomechanics of the elbow joint [1, 10]. Stable fixation is also crucial for achieving early mobilization and reducing the risk of stiffness, post-traumatic arthritis, and functional impairment [8, 11].

Traditionally, metallic implants such as titanium screws or small fracture plates have been used to stabilize radial head fractures [12, 13]. While effective, these materials may lead to hardware-related complications, including implant prominence, increased cartilage damage, local irritation, interference with imaging, and the potential need for hardware removal [13, 14]. Therefore, biodegradable implants were introduced to reduce these risks while maintaining adequate stability for bone healing [15, 16]. Polylactide pins (PP), as representatives of bioresorbable polymers, have been widely adopted in the fixation of small osteochondral or metaphyseal fragments, including radial head fractures [16–18]. Despite their advantages and widespread clinical use, polylactide pins inherently exhibit inferior mechanical strength compared to metallic implants, which may result in higher rates of secondary loss of reduction or inadequate bone healing [14, 19].

In recent years, biodegradable metals such as magnesium and zinc alloys have emerged as promising alternatives to polymer-based implants [15, 20]. Zinc implants, in particular, have attracted interest due to their moderate corrosion rate, high mechanical strength, and favorable biocompatibility profile [21–23]. Unlike polymers, zinc pins (ZP) have been reported to provide greater initial stability and avoid acidic degradation byproducts [23]. Experimental studies have suggested that zinc-based implants may even support osseointegration and bone healing while gradually resorbing, thereby eliminating the need for implant removal [24]. Our previous biomechanical study directly compared polylactide, magnesium, and zinc pins under different loading conditions in a standardized sawbones model of Mason type II radial head fractures, demonstrating promising biomechanical performance of zinc pins [25]. To further validate our previous findings, we designed the present study to evaluate the biomechanical stability under cyclic loading conditions, fracture motion, and load-to-failure characteristics of zinc and polylactide pin constructs in a paired human cadaveric Mason type II radial head fracture model, providing a more anatomically representative setting than standardized sawbones testing.

Based on the mechanical properties of zinc-based implants compared with polylactide polymers, including higher tensile strength [23, 26], we hypothesized that zinc pins would demonstrate (1) higher primary stability, (2) reduced fracture displacement unter cyclic loading, and (3) higher load-to-failure compared with polylactide pins.

## Materials and methods

The local ethics committee of the University of Freiburg approved the experimental design (protocol 25-1014-S1), and all methods were carried out in accordance with the approved guidelines. Biomechanical testing was performed using eight pairs of fresh-frozen human cadaveric radii (n = 16), consistent with general recommendations for basic cadaveric studies and the practical limitations of cadaveric research, including donor availability and cost [27]. The specimens were derived from 4 female and 4 male donors with a mean age of 46.6 years (range, 29–60 years) and a mean body mass index of 31.2 kg/m^2^ (range, 25.1–36.6 kg/m^2^).

Radial head morphology and bone density were assessed on pre-experimental computed tomography (CT) scans obtained using a Siemens SOMATOM Force scanner (Siemens Healthineers, Erlangen, Germany) with identical acquisition and reconstruction parameters for all specimens (150 kV, 12 mAs, 1.0-mm slice thickness, Br36 bone kernel reconstruction). Articular surface area (mm^2^) and diameter (mm) were measured on axial images at the level of the largest articular cross-section using circular ROIs. Subcortical bone density was determined by averaging Hounsfield unit (HU) values from three consecutive axial subcortical slices beneath the articular surface, excluding cortical bone, with identical imaging settings applied to ensure intra-study comparability.

**Fig. 1:**
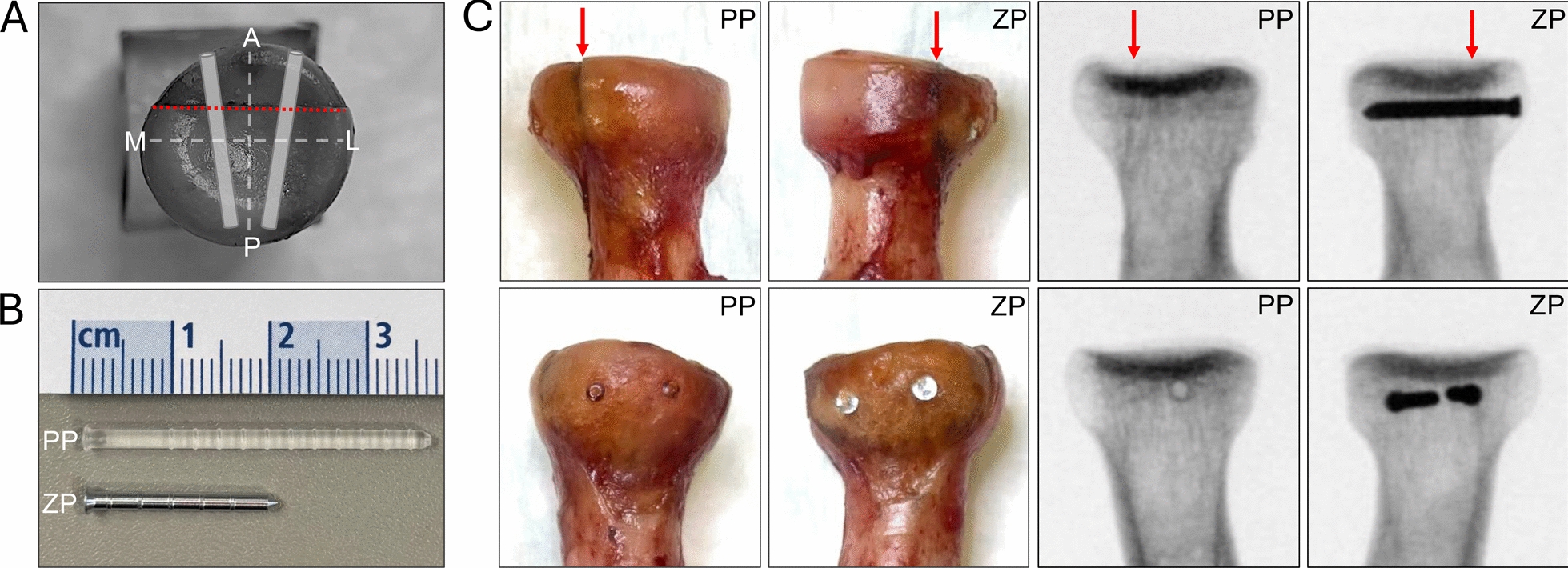
Representative specimens, fracture morphology, and implants. **A** En-face view of the radial head showing fracture plane (red dashed line) and pin configuration. **B** Biodegradable fixation implants. **C** Representative specimens with fracture lines indicated (red arrows) and radiographic control after fixation. A, anterior; M, medial; P, posterior; L, lateral. ZP, zinc pin; PP, polylactide pin

Human cadaveric specimens were prepared, and soft tissue was removed by anatomical dissection. A standardized Mason type II fracture of the radial head was created by measuring the articular surface area of every specimen and defining the exact fracture plane individually as one-third of the radial head articular surface, including the safe zone of the radial head circumference (Fig. 1A). High-precision plane osteotomies were achieved by using an adjustable specimen clamp and a water-cooled saw, type cut grinder (Model 011; Patho-service GmbH, Hamburg, Germany) with a blade thickness of 0.4 mm. The fractures ended tangentially to the radial shaft to avoid any bony support during axial or transverse loading. Fracture fixation was performed pair-by-pair by using either two zinc pins (ZP) (LiMedion GmbH, Mannheim, Germany) or two polylactide pins (PP) (PolyPIN®; Biovision, Ilmenau, Germany), each measuring 2.0 mm in diameter (Fig. 1B). Zinc pins (ZP) were composed of a zinc-silver alloy containing 3.3 wt% silver, biomechanically tested in previous studies [21, 22, 25]. Left and right radii were assigned to groups using a randomization protocol, and fracture fixation procedures were performed by the same surgeon. Both polylactide and zinc pins were implanted strictly according to the manufacturer’s manual. All pins were implanted parallel to the articular joint’s surface, in a slightly converging orientation, and the length was modified individually to ensure the tip of each pin ended subchondral (Fig. 1A, C). All osteosyntheses were radiologically controlled in 2 planes before subsequent testing (Fig. 1C). Specimens were shortened and embedded into standardized carton cuboids in polymethylmethacrylate (PMME Technovit 3040; Heraeus Kulzer GmbH, Wehrheim, Germany), with a free bone stock of approximately 5 cm to ensure adequate mounting into the testing machine, as described previously [20, 25]. Specimens were received as fresh-frozen samples and stored at − 80 °C until use. To avoid repetitive freeze–thaw cycles, each specimen underwent one experimental run after overnight thawing at room temperature. Specimen preparation, osteosynthetic construct preparation, mounting, and final biomechanical testing were then performed consecutively, with testing carried out immediately thereafter to minimize dehydration.

Testing protocols were performed by using the servo-hydraulic testing machine Amsler HC10 (Zwick/Roell, Ulm, Germany) equipped with an integrated 10 kN load cell corresponding to the standard system configuration and providing linear force measurement within the applied loading range according to manufacturer specifications.

**Fig. 2:**
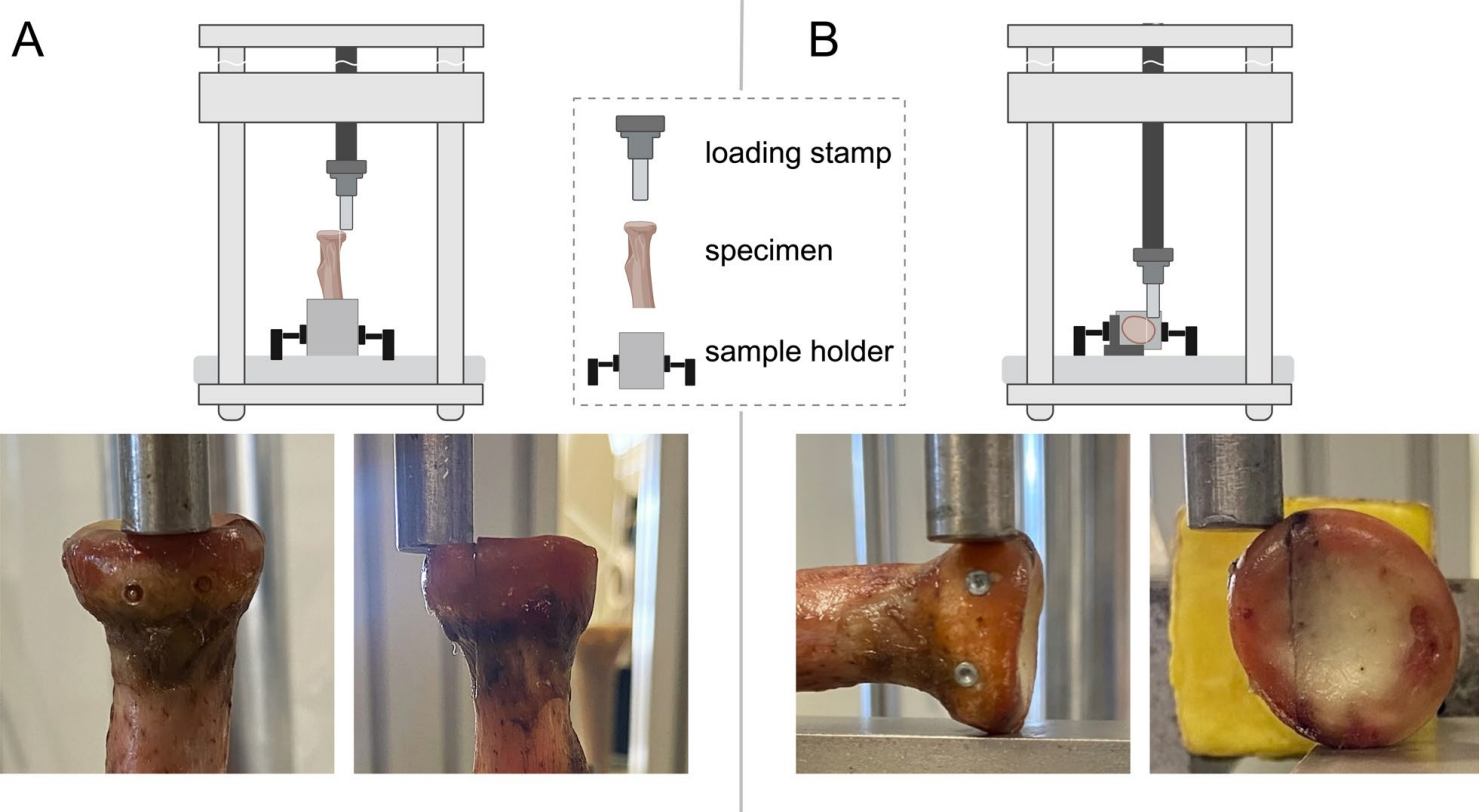
Biomechanical test setup for axial and transverse loading. Experimental setup during axial **A** and transverse **B** loading. Load was applied parallel to the fracture plane and limited to the fracture fragment. Parts of this figure were modified from [25] and created in BioRender. Maier, J. (2026) https://BioRender.com/bds6j7w

Axial and transverse loading forces were applied to the fragment through a cylindrical stamp. Cyclic loading was performed with sinusoidal forces between 15 and a maximum of 50 Newtons, mimicking forces experienced by the radial head during passive range-of-motion exercises and typical light-weight exercises during the initial postoperative period, as described in our previous study [25]. While axial loading was tested with vertically positioned specimens, transverse loading was applied to specimens positioned in their cross-axis, with a metal block as a counterpart supporting the radial head except for the fragment (Fig. 2). In both settings, loading was parallel to the fracture plane, and any support to the fragment was prevented. As described previously, our testing protocol started with 10 transverse cyclic loads between 15 and 50 N at 0.1 Hz, testing the stability of the anatomic reduction and the restored radial head circumference [25]. Once transverse stability was ensured, subsequent repetitive loadings to the anterior fragment occur mainly during elbow flexion, simulated by subsequent axial loading at 0.1 Hz between 15 and 50 N for a total of 1,000 cycles. Finally, load-to-failure testing was carried out with a continuous increase of the axial load (2 N per second) until > 2 mm fracture displacement and subsequent construct failure occurred. Displacement measurements were derived from crosshead displacement recorded by the testing machine. Due to the small fracture fragment size in radial head fractures, additional local measurement systems at the fracture site were not technically feasible without interfering with construct integrity. The testing protocol, as well as real-time fracture displacement (mm) and loading force (N), were monitored by using the TestXpert® II software (Zwick, Ulm, Germany).

To address the research questions, the following outcome parameters were measured:

1. *Construct stiffness (kN/mm)* to evaluate primary stability and fixation strength of the constructs.
2. *Cyclic micromotion (mm)*, defined as the reversible peak-to-peak displacement of the fracture fragment during sinusoidal loading, and *cyclic migration (mm)*, defined as the progressive irreversible drift of the fracture fragment relative to its initial position between the first and last ten cycles, to assess fracture displacement and construct loosening under repetitive loading.
3. *Load at 2 mm dislocation (N)*, defined as the force at which fracture displacement exceeded 2 mm, and ultimate *load to failure (N)*, defined as the maximum load sustained before complete mechanical failure of the fixation construct with abrupt loss of load-bearing capacity due to implant breakage or gross fragment displacement, to determine resistance to maximum forces and clinical failure.

Data were collected and synthesized using Microsoft Excel (v16.94; Microsoft Corp., Redmond, WA, USA). Statistical analyses and data visualization were performed using R statistical software (v4.5.1; R Foundation for Statistical Computing, Vienna, Austria) with the *stats*, *dplyr*, *tidyr*, and *purrr* packages. Biomechanical comparisons between zinc pin (ZP) and polylactide pin (PP) fixation were conducted using a paired design, with contralateral radii from the same donor analyzed as matched pairs. Normality of paired differences was assessed using the Shapiro–Wilk test. As normality was confirmed for all parameters (all *p* > 0.05), two-sided paired t-tests were applied. To account for multiple testing across biomechanical outcomes, Holm correction was performed, and adjusted *p*-values were used to determine significance. Data are presented as mean ± standard deviation, and *p* < 0.05 was considered statistically significant. Exploratory Pearson correlation analyses were conducted to assess associations between bone density (HU) and biomechanical parameters, analyzed separately within each fixation group.

## Results

Pre-experimental computed tomography showed no differences between the two groups with respect to bone density (ZP vs. PP: 159 ± 44 vs. 158 ± 39 HU; *p* = 0.966) or subchondral articular surface area (ZP vs. PP: 4.29 ± 0.91 vs. 4.23 ± 0.95 cm^2^; *p* = 0.367), confirming adequate comparability of paired specimens (Table 1).

**Table 1:** Overall results of biomechanical testing

Outcome parameter	Group (n)	Mean (± SD)	95% CI	*P* value*	P adj
Axial stiffness (kN/mm)	ZP (6) PP (6)	0.77 ± 0.20 0.33 ± 0.05	[0.56; 0.98] [0.28; 0.38]	0.005	0.028
Transverse stiffness (kN/mm)	ZP (8) PP (8)	0.21 ± 0.06 0.20 ± 0.09	[0.16; 0.26] [0.12; 0.28]	0.736	0.946
Axial micromotion (mm)	ZP (6) PP (6)	0.05 ± 0.01 0.10 ± 0.02	[0.04; 0.06] [0.08; 0.12]	0.010	0.042
Axial migration (mm)	ZP (6) PP (6)	0.17 ± 0.09 0.20 ± 0.10	[0.08; 0.26] [0.10; 0.30]	0.473	0.946
Load at 2 mm dislocation (N)	ZP (8) PP (8)	161 ± 37 112 ± 60	[130; 192] [62; 162]	0.039	0.116
Load at construct failure (N)	ZP (8) PP (8)	190 ± 43 116 ± 62	[154; 226] [64; 168]	0.008	0.042
Bone Density (HU)	ZP (8) PP (8)	159 ± 44 159 ± 39	[122; 196] [126; 192]	0.966	
Subchondral surface area (cm^2^)	ZP (8) PP (8)	4.29 ± 0.90 4.22 ± 0.95	[3.54; 5.04] [3.43; 5.01]	0.367	

*ZP vs. PP comparison. P values were adjusted for multiple testing across biomechanical outcome parameters using the Holm correction. *SD* Standard deviation; *CI* Confidence interval; *kN* kilonewton; *N* Newton; *mm* millimeter; *cm* centimeter; *HU* Hounsfield Unit; *ZP* Zinc Pin; *PP* Polylactide Pin

**Fig. 3:**
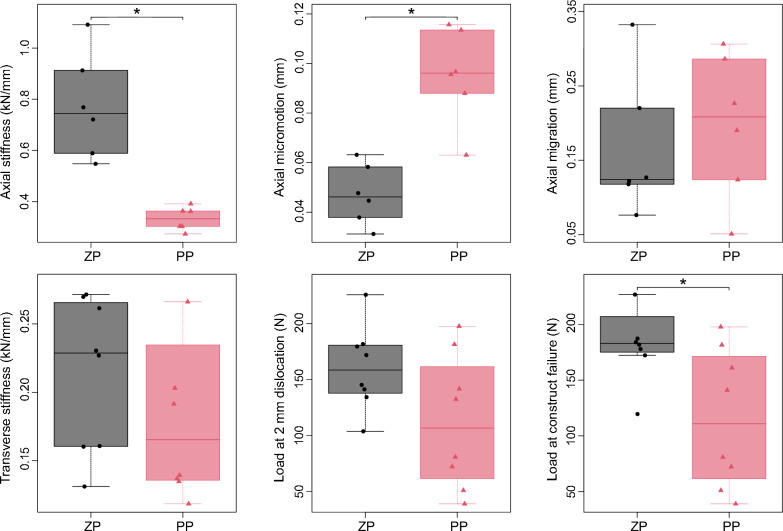
Biomechanical comparison of zinc pin (ZP) and polylactide pin (PP) fixation in paired human cadaveric radii. Boxes indicate the interquartile range with median; whiskers represent the minimum and maximum values; individual data points are shown. * Holm-adjusted p < 0.05

Cyclic transverse loading revealed sufficient stability of all constructs, without any relevant fracture dislocations or differences in transverse stiffness between ZP and PP constructs (0.21 ± 0.06 vs. 0.20 ± 0.09 kN/mm; *p* > 0.05; Table 1 and Fig. 3).

However, two PP constructs failed prematurely during subsequent cyclic axial loading, at the first and 167th cycles, respectively. Due to early mechanical failure of these PP constructs, the corresponding pairs were excluded from all axial loading analyses, leaving 6 evaluable paired specimens for these outcome parameters. The axial load measured at construct failure was defined as the load-to-failure in these specimens, allowing subsequent failure-load analyses of all eight pairs.

Axial stiffness was higher in the ZP group compared with the PP constructs (0.77 ± 0.20 vs. 0.33 ± 0.05 kN/mm; p-adj. = 0.028). In addition, axial cyclic micromotion was lower following fracture fixation with ZP (0.05 ± 0.01 vs. 0.10 ± 0.02 mm; p-adj. = 0.042). Although ZP constructs showed higher primary stability with lower micromotion at the fracture site, no difference was observed in axial cyclic migration between both fixation groups (0.17 ± 0.09 vs. 0.20 ± 0.10 mm; p-adj. > 0.05), as shown in Table 1 and Fig. 3.

Load-to-failure testing under increasing axial load (2 N/s) demonstrated higher loads at 2 mm fracture displacement in the ZP group (161 ± 37 vs. 112 ± 60 N; p-adj. = 0.116), even though statistical significance was not reached after correction for multiple testing. However, maximum loads at construct failure were higher for ZP constructs compared to the PP group (190 ± 44 vs. 116 ± 62 N; p-adj. = 0.042; Table 1 and Fig. 3).

**Fig. 4:**
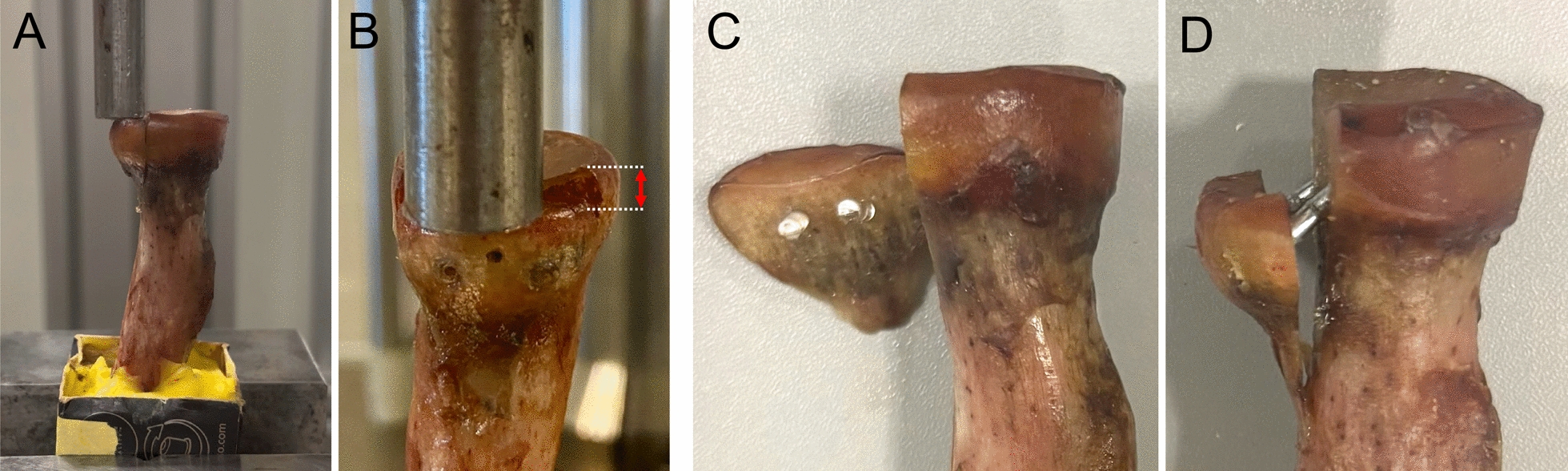
Load-to-failure testing and construct failure. **A** Clinically relevant fracture displacement was defined at ≥ 2 mm. **B** Representative specimens after complete construct failure, including implant breakage in a polylactide pin construct (**C**) and failure of a zinc pin construct (**D**)

Macroscopic analysis of failure patterns showed complete implant breakage in polylactide pins under maximum loading, whereas zinc pins demonstrated bending of the implants or cortical breakage, leading to significant fracture displacement (Fig. 4).

**Fig. 5:**
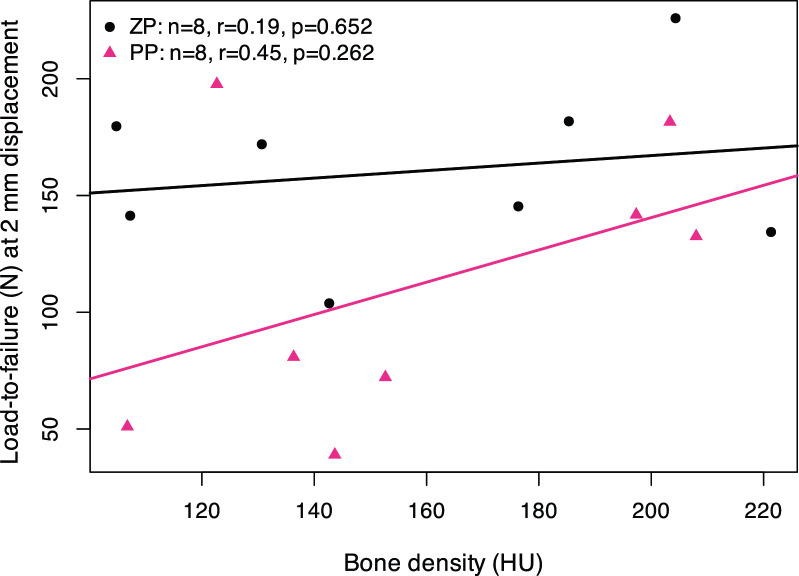
Correlation analysis of bone quality and biomechanical performance. Scatter plot showing the exploratory association between bone density (HU) and load-to-failure at 2 mm fracture displacement by fixation type. Symbols represent individual specimens; solid lines indicate group-specific linear regression fits. Pearson correlation coefficients (r) and p-values are shown. ZP, zinc pin; PP, polylactide pin; HU, Hounsfield unit

The relationship between bone density (HU) and biomechanical performance was explored using Pearson correlation analysis, stratified by fixation type (ZP vs. PP), focusing on load-to-failure at 2 mm fracture displacement as the clinically relevant endpoint. ZP constructs demonstrated a weak positive correlation with bone density (r = 0.19, *p* = 0.652, Fig. 5), whereas polylactide pin constructs showed a moderate positive correlation (r = 0.45, *p* = 0.262; Fig. 5). However, neither association reached statistical significance, indicating no clear dependency of construct performance on bone density within the investigated range.

## Discussion

The present study provides a biomechanical comparison of biodegradable zinc and polylactide pins for the fixation of displaced Mason type II radial head fractures using paired human cadaver radii. Fracture fixation with zinc pins demonstrated superior mechanical stability compared with polylactide pins under axial cyclic testing and load-to-failure. Constructs stabilized with zinc pins exhibited lower micromotion at the fracture site and higher resistance to progressive loading, suggesting a reduced risk of secondary loss of reduction and more favorable mechanical conditions during the early fracture healing phase. These findings are consistent with our previous study in artificial composite sawbones [25], and underline the higher intrinsic strength and stiffness of zinc pins compared with polylactide pins [23]. Further, our data support the hypothesis that biodegradable metallic alloys may overcome mechanical limitations of polymer-based implants [10, 19]. From a clinical perspective, sufficient primary stability is critical in Mason type II fractures to allow early elbow motion while preventing fragment displacement and maintaining adequate fracture fixation during early consolidation.

Overall, both zinc and polylactide pins demonstrated stable construct behavior in our tests. No significant differences in implant loosening were detected, as reflected by comparable axial migration during cyclic loading. This was also shown in a previous study, where polylactide pins did not show a higher rate of implant loosening than headless titanium screws [14]. In addition, failure loads at the 2 mm fracture displacement did not differ significantly between the groups. These results indicate sufficient resistance to early fragment dislocation for both fixation methods. This is also in line with previous clinical studies on polylactide pin fixation in radial head fractures and support its clinical adequacy under physiologic loading conditions [16, 17, 28]. While zinc pins may provide a greater mechanical safety margin, the present data do not suggest mechanical insufficiency of polylactide pins for their intended clinical application. Rather, the superior primary stability and resistance to axial loads of ZP suggest a potentially broader safety margin for functional rehabilitation protocols, whereas polylactide pins remain a biomechanically sound option for appropriately selected cases [19, 29]. Nevertheless, we exhibited early loss of reduction under axial loading only in polylactide pin constructs. Thus, the inferior mechanical performance and early construct failures may necessitate more cautious postoperative care and may partly explain reported cases of secondary displacement with polymer-based implants in clinical practice [16, 19]. At this point, it should be noted that the applied cyclic loading protocol (15–50 N) primarily reflects protected early postoperative conditions rather than full physiological loading during unrestricted daily activities. Although this range was selected to simulate passive motion and light functional loading during early rehabilitation, higher joint reaction forces occur during active elbow use. Therefore, the present findings should mainly be interpreted in the context of early postoperative mechanical stability.

By using a cadaveric model, this work extends our previous investigations seen in sawbones [25] while offering a more physiologically relevant assessment that accounts for interindividual variability in bone morphology and quality. The use of paired radii from the same donors minimizes confounding effects related to bone density, geometry, and cortical thickness. This is particularly relevant in the radial head, where subchondral bone quality and fragment size have been reported to substantially influence fixation stability [2, 14, 30–32]. Importantly, this analysis was performed in a non-osteoporotic donor population, as all specimens were derived from relatively young donors (< 60 years of age). Consequently, the range of bone quality was limited, and no osteoporosis model was investigated. Although a trend toward higher failure loads with increasing bone density was observed, no statistically significant associations were identified within either fixation group in this paired-sample cohort. Within this context, bone density did not appear to be a primary determinant of failure behavior at the clinically relevant 2 mm displacement threshold. Therefore, the observed biomechanical differences between zinc and polylactide pin fixation are more likely attributable to implant-related characteristics rather than variations in bone quality.

Several limitations should be considered when interpreting our results. First, this study was limited to biomechanical analyses and did not include biological assessment. Thus, conclusions about in vivo degradation behavior, local tissue response, or long-term outcomes cannot be drawn. Second, although the loading protocol was designed to approximate physiologic forces, our fracture model does not fully replicate the complex, multidirectional loading and muscular forces at the elbow joint. Third, the sample size, while comparable to other cadaveric biomechanical studies [14, 20, 33–35], limits statistical power, particularly for detecting smaller effect sizes. In addition, construct failures in the PP group reduced the effective sample size for selected analyses and thereby further limited statistical sensitivity for these endpoints. Consequently, non-significant findings should be interpreted cautiously, particularly where only small between-group differences were observed. While a larger sample size would have been preferable, cadaveric specimen availability was restricted by ethical considerations, donor availability, and cost. A further limitation is that the standard 10 kN load cell of the testing system was used despite the relatively low applied force range (5–200 N). However, all applied forces remained above the system’s resolution threshold, and as the study focused on relative differences between groups under identical testing conditions, any potential systematic measurement deviation is unlikely to have influenced the comparative results.

## Conclusion

This biomechanical study demonstrates that zinc pins provide superior biomechanical stability compared with polylactide pins for the fixation of Mason type II radial head fractures in a human cadaveric fracture model. The overall higher primary stability, reduced fracture-site motion, and increased resistance to axial loading and secondary displacement extend our previous findings obtained in standardized composite bone models. Together, these results indicate robust construct performance across anatomically variable human radial heads, with no significant susceptibility to variations in bone density within the investigated range. These results support further translational and clinical investigations of zinc-based biodegradable implants. Future studies should focus on their in vivo performance, safety profile, and potential to improve clinical outcomes while preserving the advantages of biodegradability.

## Data Availability

The datasets generated and/or analyzed during the current study are available from the corresponding author on reasonable request.
